# The Role of Red Blood Cells in Cholesterol Accumulation and Atherosclerotic Plaque Instability: A Perspective on Atherosclerosis

**DOI:** 10.2174/011573403X358572250128104335

**Published:** 2025-02-11

**Authors:** Reza Mohebbati, Mohammad Amin Momeni-Moghaddam

**Affiliations:** 1 Department of Physiology, Faculty of Medicine, Gonabad University of Medical Science, Gonabad, Iran;; 2 Department of Nutrition and Biochemistry, Faculty of Medicine, Social Determinants of Health Research Center, Gonabad University of Medical Science, Gonabad, Iran

**Keywords:** Red blood cells, cholesterol, plaque, atherosclerosis, cardiovascular, angiogenesis

## Abstract

Atherosclerosis stands as the primary cause of CVD, characterized by the accumulation of cholesterol deposits within macrophages in medium and large arteries. This deposition promotes the proliferation of specific cell types within the arterial wall, gradually narrowing the vessel lumen and impeding blood flow. Intra-plaque hemorrhages are recognized as critical events in atherosclerotic plaques, leading to the deposition of red blood cells (RBCs) and the release of hemoglobin (Hb). Approximately 40% of high-risk plaques exhibit intra-plaque hemorrhage. Recent studies have demonstrated that intra-plaque hemorrhage is closely linked to plaque progression and increased vulnerability, establishing it as a critical factor in the development of acute clinical symptoms associated with atherosclerosis. The presence of RBC membranes within atherosclerotic plaques contributes significantly to lipid accumulation, indicating a pivotal role in plaque instability. Upon RBC degradation, cholesterol from both the membrane and its interior can profoundly impact atherosclerotic plaque development. Considering that red blood cells (RBCs) can contribute to the excretion of cholesterol through the hepatobiliary system alongside HDL, and given that elevated cholesterol levels are a risk factor for the development and progression of atherosclerotic plaques, RBCs may play a protective role in cardiovascular health. However, when bleeding occurs within a plaque, RBCs that are trapped in the plaque environment, an environment rich in oxidant compounds, can rupture. The cholesterol released from these ruptured RBCs can significantly promote inflammatory reactions. This study aims to explore the inconsistent role of RBCs and their cholesterol content in the progression of atherosclerotic plaques.

## 1 INTRODUCTION

Cardiovascular diseases (CVD) represent a significant global threat to health [1, 2]. It is predicted that by 2030, over 23.3 million people will die annually from CVD [3]. Atherosclerosis stands as the primary cause of CVD [4, 5], characterized by the accumulation of cholesterol deposits within macrophages in medium and large arteries. This deposition promotes the proliferation of specific cell types within the arterial wall, gradually narrowing the vessel lumen and impeding blood flow. The development of atherosclerosis is influenced by a multitude of environmental and genetic factors, including age, sex, obesity, smoking, blood pressure, and diabetes mellitus [6-8]. Research indicates that oxidative stress plays a crucial role in the pathophysiology of atherosclerosis [9, 10]. Oxidative stress results from an imbalance between the production and accumulation of reactive oxygen species (ROS) in tissues and cells. When ROS production overwhelms the antioxidant defense system, oxidative stress ensues [11, 12]. Oxidized red blood cells (RBCs) contribute to various cardiovascular complications, including atherogenesis, hypertension, cardiovascular diseases, and stroke. Studies have demonstrated that RBCs are present within atherosclerotic plaques. The presence of RBCs in these plaques can be attributed to two distinct mechanisms, both of which are associated with plaque instability. The first mechanism involves the entry of RBCs into the plaque following the disruption of the fibrous cap [13].

Plaque disruption is a critical event that contributes to the clinical manifestations of atherosclerosis. Mature atherosclerotic plaques can be categorized into two types: stable and vulnerable, based on their susceptibility to disruption. Stable plaques are characterized by a small lipid core and a thick fibrous cap, while vulnerable plaques have a significant lipid core and a thin fibrous cap. These morphological differences indicate that vulnerable plaques are structurally weaker and more likely to rupture under the physical stresses of blood flow [6]. The second mechanism is intra-plaque hemorrhage, which occurs due to the increased fragility of microvessels within the plaque. These new capillaries typically consist of a single layer of endothelial cells resting on a basement membrane with smooth muscle cells. These fragile vessels are prone to rupture, leading to the leakage of erythrocytes into the plaque environment [13]. Intra-plaque hemorrhages are identified as vulnerable events in atherosclerotic plaques, leading to the deposition of RBCs and the release of hemoglobin (Hb). The presence of RBC membranes within atherosclerotic plaques significantly increases lipid accumulation, highlighting their role in plaque instability [14] . This study aims to review the role of RBC in the development of atherosclerotic plaques.

Databases such as PubMed, Web of Science and Scopus were considered for research. The search terms were “Red blood cells”, “Plaque”, “Atherosclerosis”, “Cholesterol”, “Cardiovascular” and “Angiogenesis” alone and together. The full-text English articles were selected and reviewed from Oct 2001 to Oct 2024. Other irrelevant and presentation abstracts were excluded.

## 2 ATHEROSCLEROTIC PLAQUE FORMATION

Pathogenic events in atherogenesis involve endothelial dysfunction and activation, monocyte/macrophage adhesion, activation and migration, local oxidative stress, lipid deposition, extracellular matrix (ECM) synthesis, smooth muscle cell (SMC) migration and proliferation, and neovascularization [15]. Atherosclerotic plaques develop within the arterial wall, which consists of three layers. The innermost layer, the intima, contains a single layer of endothelial cells that regulate processes such as thrombosis, vascular tone, and leukocyte passage. The middle layer, or media, is predominantly composed of smooth muscle cells (SMCs). The outer layer, or adventitia, includes elastin, smooth muscle cells, fibroblasts, and collagen [6]. Endothelial cell exposure to high levels of LDLs leads to dysfunction and lipid deposition in the intima. Chronic exposure to other pathogenic factors like infectious diseases, hypertension, diabetes, stress, and smoking further damages the endothelium [3]. These lipoproteins undergo oxidation, triggering inflammatory responses [16]. Modified LDLs enhance pro-inflammatory reactions, recruiting and mobilizing innate immune cells, such as monocytes, which differentiate into macrophages under the influence of macrophage colony-stimulating factor [3]. Once differentiated, macrophages uptake oxidized LDLs *via* receptors like CD36, becoming foam cells filled with lipids [3, 5]. As atherosclerosis progresses, smooth muscle cells migrate from the media to the intima in response to growth factors and cytokines secreted by activated endothelial and macrophage cells. Within the intima, these cells proliferate and produce ECM components, contributing to fibrous cap formation. Many of the lipid-laden macrophages undergo apoptosis in the early stages, cleared by M2 macrophages through efferocytosis. However, excessive apoptotic cell uptake can lead to endoplasmic reticulum stress, impairing efferocytosis and resulting in macrophage death, release of lipids, pro-inflammatory/thrombotic mediators (*e.g*., tissue factor, MMPs), and ECM degradation. MMPs degrade ECM scaffolds, including the fibrous cap, rendering the plaque susceptible to rupture. Plaque vulnerability is also characterized by reduced SMC content and the formation of immature, leaky vessels within the necrotic core [3]. Additionally, macrophages residing within advanced plaques contribute to plaque destabilization by secreting cytokines, proteases, and thrombotic factors [16].

## 3 RBCS CHOLESTEROL

The RBC membrane, a simplified plasma membrane, contains fewer components compared to other cell types. Its primary components are phospholipids and cholesterol, with RBC membranes being 1.5 to 2 times richer in cholesterol than membranes of other body cells, predominantly in free form [13, 17, 18]. Interestingly, RBC cholesterol levels are comparable to those found in plasma lipoproteins, and RBCs are involved in reverse cholesterol transport to hepatocytes [19, 20]. Research in apoAI-deficient mice with dysfunctional HDL has shown that RBCs carry a significant fraction of blood cholesterol and contribute to cholesterol reverse transport to feces, indicating a non-HDL-mediated peripheral cholesterol uptake pathway. This pathway was impaired in apoAI-deficient mice with anemia, highlighting the importance of this alternative route [21]. While the well-established pathway for cholesterol reverse transport involves HDL metabolism, studies suggest the existence of additional pathways. In apoAI-deficient mice, despite the absence of HDL, cholesterol reverse transport to the liver was maintained, with RBC cholesterol levels being twice as high as plasma levels [22]. Upon passing through the liver, RBCs transfer cholesterol to sinusoidal endothelial cells, facilitating its entry into hepatocytes. Additionally, studies by Ohkawa demonstrated cholesterol transfer between RBCs and lipoproteins following incubation of RBCs with autologous plasma. This exchange involves the movement of cholesterol from RBCs to HDL and from LDL to RBCs [23]. Furthermore, research by Shao-Jui Lai showed that RBCs can receive cholesterol from THP-1 macrophages mediated through HDL [20]. Clinical studies have linked RBC levels with HDL-C and the prevalence of coronary artery disease (CAD). Higher RBC levels correlate with reduced CAD risk, particularly in individuals with lower HDL-C levels [21]. Conversely, studies in individuals with anemia have shown lower lipid profiles, underscoring the significant role of RBCs in cholesterol metabolism [24, 25]. Therefore, RBCs play a crucial role similar to HDL in cholesterol reverse transport pathways. Reduced RBC levels may thus pose a risk factor for cardiovascular disease, based on these findings.

Additionally, studies indicate that elevated LDL cholesterol levels can lead to excessive cholesterol transfer to the membranes of red blood cells (RBCs), resulting in a condition known as spur-cell anemia. This underscores the relationship between blood cholesterol levels and RBC characteristics [26]. Several factors influence the transfer of plasma cholesterol to erythrocytes. These factors include the cholesterol content in lipoprotein particles, the number and distribution of transfer sites on the RBC membrane, and the structure and composition of the membrane, particularly the sphingomyelin (SM) content and the activity of lecithin cholesterol acyltransferase (LCAT) associated with the erythrocyte. Statin treatment has been shown to significantly reduce plasma cholesterol levels within one month, with RBC membrane cholesterol decreasing and stabilizing after six months in the same patients. The lipid-rich RBC membrane likely contributes to the cholesterol content in atherosclerotic plaques [13]. In addition to membrane cholesterol, Nikolić's study on RBC lipid content revealed the presence of cholesterol and phospholipids tightly bound to hemoglobin, forming a complex called lipid-hemoglobin (Hb-Ch). This complex binds approximately two molecules of cholesterol and phospholipids to each Hb tetramer, constituting about 0.2% to 55% of total hemoglobin. There is a positive correlation between Hb-Ch and HDL cholesterol levels, indicating a direct influence of plasma lipoprotein metabolism on Hb-Ch formation. Hb-Ch is believed to represent a novel form of circulating cholesterol that aids in the removal of excess unesterified cholesterol from circulation. Thus, RBCs may serve as a frontline defense mechanism against excessive free cholesterol in circulation [27].

## 4 ROLE OF RBC CHOLESTEROL IN THE LIPID CORE OF ATHEROSCLEROTIC PLAQUES

The lipid core of atheromas is enriched with extracellular lipids, including free cholesterol, esterified cholesterol, and oxidized lipoproteins. Research suggests that the free cholesterol within the necrotic core of advanced plaques may originate from sources other than macrophages, possibly from erythrocytes due to their high membrane cholesterol content [13, 28-31]. Histopathological studies by Arbustini *et al.* on atherosclerotic plaques of pulmonary arteries demonstrated that glycophorin derived from RBC membranes is a significant component of these plaques, highlighting the role of erythrocyte membrane compounds in atherogenic processes [31]. Kolodgie's study further observed RBC membrane remnants within advanced coronary atheromas. Injecting erythrocytes into lesions in rabbits induced plaque formation containing free cholesterol, lipids, and iron, contrasting with control lesions exhibiting lower free cholesterol levels [32]. Clinical studies have shown significantly higher total cholesterol levels in erythrocyte membranes (CEM) in patients with acute coronary syndrome (ACS) compared to those with chronic stable angina (CSA), suggesting CEM as a potential marker for atheromatous plaque growth and vulnerability [33]. Furthermore, increased RBC presence within plaques correlates with elevated plaque lipid content, enhanced macrophage permeability, and superoxide production, elevated levels of interleukin-1 and IFN-γ, and reduced fibrous cap thickness [34]. High-fat diets induce changes in RBCs, including increased membrane cholesterol content, accelerating the removal of RBC cholesterol by macrophages and basal cell formation [35]. These changes indicate that RBC presence not only increases plaque lipid content but also promotes inflammation and cytokine release at atherogenic lesion sites. These changes indicate that the presence of red blood cells in atherosclerotic plaques not only increases their lipid content but also heightens inflammation at the sites of atherogenic lesions by elevating pro-inflammatory cytokines.

The question arises: how do erythrocytes come to be present in atherosclerotic plaques and contribute to increased cholesterol levels in these lesions? Intraplaque bleeding signifies the presence of red blood cells (RBCs) in atherosclerotic plaques, defined as the accumulation of blood components within these plaques. Intraplaque hemorrhage is increasingly recognized as a critical mechanism through which atherosclerotic plaques can rapidly progress and significantly increase in total volume [36]. Bleeding within the plaque can result from fibrous cap rupture, disruption of endothelial integrity, or dysfunction and leakage from small vessels within the plaque [37]. Another proposed mechanism involves neovascularization leading to vessel rupture. Eryptosis, a process where RBCs undergo cell death, can also contribute to bleeding within the plaque due to cholesterol-induced stiffening of the RBC membrane, reducing its deformability. RBCs with stiff membranes are less stable and more prone to mechanical slippage, particularly in narrow capillaries. Pathologically elevated cholesterol levels further increase membrane cholesterol content, decreasing RBC stability and deformability [38]. Upon entry into the plaque environment, RBCs are exposed to increased free radicals, damaging their sensitive membranes and releasing their contents, including cholesterol. RBCs can easily lyse in the plaque's microenvironment, releasing iron and heme groups. Plaques at higher risk feature large necrotic cores with thin fibrous caps. The heightened pro-inflammatory environment within the plaque increases protease activity, further increasing the risk of fibrous cap rupture [39]. Additionally, disruption and rupture of small vessels within the plaque, formed during angiogenesis, can also facilitate RBC entry into these areas [40].

## 5 HYPOXIA AND INFLAMMATION ARE TWO KNOWN STIMULI FOR ANGIOGENESIS

One proposed mechanism for the infiltration of red blood cells (RBCs) into atherogenic plaques involves the rupture and leakage of vessels formed during the angiogenesis process within the plaques. Angiogenesis is crucial for tissue proliferation and repair. Physiological angiogenesis acts as a defense mechanism in response to tissue hypoxia, restoring vessel wall homeostasis [41].

Endothelial damage in arteries due to factors such as abnormal shear stress triggers the migration and proliferation of vascular smooth muscle cells (VSMCs), leading to intimal thickening. This condition induces hypoxia, stimulating angiogenesis of the vasa vasorum [42]. Adventitial neovascularization in response to angiogenic stimuli is the widely accepted mechanism for new vessel formation [43]. Lumenal endothelial cells may also contribute to new vessel formation by recruiting and differentiating progenitor cells within the plaque. However, vessels within plaques differ anatomically from normal vessels and exhibit altered responses to stimuli. These dysmorphic vessels feature a discontinuous basement membrane, reduced endothelial cell tight junctions, and sparse smooth muscle cells or pericytes, rendering them leaky and prone to rupture [44].

In a healthy state, oxygen and nutrients diffuse from the intravascular space to the intimal and medial cells, while the outer layers of the media and adventitia are nourished by the vascular network of the vasa vasorum. As plaques enlarge, increased oxygen demand by metabolically active cells like macrophages creates a hypoxic microenvironment within the lesion. Neovascularization is a prominent feature of advanced atherosclerotic plaques. However, evidence suggests that neovascularization can also occur in early atherosclerotic lesions, particularly when the intimal thickness exceeds the oxygen diffusion distance (approximately 200-250 μm) [44-46].

In advanced atherosclerotic plaques, hypoxia, coupled with macrophage-driven inflammation and oxidative stress, facilitates the oxidation of LDL to ox-LDL. These processes lead to the persistent release of vascular endothelial growth factor (VEGF), promoting neoangiogenesis. New vessels originating from the vasa vasorum extend from the adventitia into the intima of medium and large arteries. These vessels are characterized by their disorganization, lack of smooth muscle cells, and deficient endothelial gap junctions, rendering them prone to rupture and facilitating the entry of erythrocytes into the plaque [38, 46-49].

Hypoxia is a well-established stimulus for angiogenesis. Metabolic changes from aerobic to anaerobic states, marked by ATP and glucose depletion and lactate accumulation, occur within atheromas. Recent studies have demonstrated that both intermittent and sustained hypoxia accelerate atherosclerosis in mice deficient in apolipoprotein E (apoE). The hypoxia-inducible factor-1 (HIF-1) pathway serves as the principal mediator of hypoxia's biological effects. HIF-1 functions as a heterodimer composed of HIF-1α and HIF-1β subunits. While HIF-1β levels remain stable, HIF-1α levels are regulated by oxygen availability. Under normoxic conditions (normal oxygen levels), HIF-1α subunits undergo hydroxylation by Fe^2+^-dependent prolyl hydroxylases (PHD), leading to their ubiquitination and subsequent degradation *via* the 26-S proteasome system. In contrast, hypoxic conditions render PHD inactive, preventing HIF-1α degradation. Consequently, stabilized HIF-1α forms a heterodimer with HIF-1β, translocates to the nucleus, and binds to hypoxia-responsive elements (HREs), initiating the transcription of target genes [44, 50].

In addition to hypoxia, inflammation serves as a potent inducer of angiogenesis by promoting the synthesis of various angiogenic factors. During acute inflammation, pro-angiogenic molecules increase vascular permeability and facilitate leukocyte infiltration into plaques, thereby sustaining chronic inflammation. The newly formed vessels within plaques are immature and inherently leaky, allowing infiltration of inflammatory cells and the influx of blood constituents, including red blood cells and platelets [51].

Recent research has highlighted the role of reactive oxygen species (ROS) in both physiological and pathological angiogenesis, particularly under conditions of oxidative stress. ROS activates the HIF-1/VEGF pathway, which is a key mechanism in ROS-mediated angiogenesis [52]. Vascular endothelial growth factor (VEGF) is recognized as a potent angiogenic growth factor in various physiological and pathological contexts. Moreover, toll-like receptors (TLRs) have been implicated in ROS-mediated angiogenic responses. Activation of different TLRs (such as TLR2, TLR3, TLR4, and TLR2/6) can stimulate angiogenesis through both HIF-1/VEGF-dependent and non-HIF-1/VEGF-dependent pathways. For instance, TLR4 activation by lipopolysaccharide triggers the HIF-1 pathway, whereas TLR2 activation by endogenous ligands induces angiogenic responses independent of VEGF, such as through the production of oxidized phospholipids [44].

ROS also contributes to angiogenesis by generating lipid oxidation products, including oxidized phospholipids, which accumulate in significant quantities within atherosclerotic lesions. These products play a role in promoting angiogenesis and influencing the progression of vascular pathology associated with atherosclerosis.

## 6 ERYTHROPHAGOCYTOSIS

Intraplaque (IP) angiogenesis is a key feature of advanced atherosclerotic plaques. As IP vessels are fragile, red blood cells are released and phagocytosed by macrophages (erythrophagocytosis), leading to high intracellular iron content, lipid peroxidation and cell death [53] . The accumulation of intracellular hemoglobin and iron after erythrophagocytosis may act as a catalyst in the formation of free radicals and contribute to lipid peroxidation, Ox-LDL generation, and cell death [26]. In addition, the erythrocytes inside the plaque contain iron and hemoglobin and may play a role in the formation of foamy cells. Erythrocyte cell surface compounds can be detected by macrophage scavenger receptors. Glycophorin A can act as ligands for scavenger receptors. These observations suggest that erythrocytes may play a major role in foam cell formation, lipid core development, and thus, plaque instability [13].

Increased RBC lipid peroxidation associated with decreased expression of glutathione-reducing proteins and increased entry of RBCs into plaques through damaged endothelium leads to erythrophagocytosis by macrophages [54].

## 7 INFLAMMATION THROUGH CHOLESTEROL

Atherosclerosis is a chronic inflammatory disease. Genetic studies have shown that a variety of genetic variants in inflammatory signaling pathways can lead to atherosclerosis. Patients with atherosclerosis have higher levels of inflammatory markers such as C-reactive protein (CRP) and interleukin-1β (IL-1β) compared to normal subjects [55]. Red blood cells with excessively stiff membranes are less stable and prone to slippage by mechanical forces, especially when passing through narrow capillaries. Red blood cells that were oxidized are phagocytosed by macrophages. Therefore, there is a rapid accumulation of cholesterol in the macrophage, because the red blood cell is one of the cells in the body with the highest amount of membrane cholesterol [27]. Following RBC lysis in the inflammatory environment of atherogenic plaque, these cells are lysed and their contents, including cholesterol, are placed inside the plaque. Disruption of HDL transport function can lead to extracellular accumulation of free cholesterol. Free cholesterol saturation can lead to cholesterol crystallization with cell death and intimal damage. Cholesterol crystals initiate inflammation *via* the NLRP3 inflammasome, which leads to the production of interleukin-1β (IL-1β), which induces C-reactive protein. Finally, crystals growing from within the plaque and associated inflammation destabilize the plaque [56]. It is also consistent that lowering cholesterol reduces pro-inflammatory responses to multiple stimuli against multiple TLRs. Macrophages are anti-inflammatory with less cholesterol and thus contribute to the anti-inflammatory action of statins [57] . Cholesterol accumulation in macrophages treated with LDL or modified cholesterol crystals leads to activation of the NLRP3 inflammasome as a result of increased membrane-to-cholesterol transport. It is widely accepted that lysosomal damage by cholesterol crystals represents a major mechanism linking excessive cholesterol in macrophages to inflammasome activation in atherosclerosis [58].

Inflammation plays an important mediating role in all stages of atherosclerosis. Over the past 20 years, it has been recognized that in addition to pathogen-associated molecular patterns (PAMPs), several endogenous molecules, called damage-associated molecular patterns (DAMPs), can activate cellular receptors, which ultimately lead to inflammation. One study showed that cholesterol crystals act as DAMPs and can activate the NLRP3 inflammasome in macrophages. The activation of NLRP3 by cholesterol crystals leads to the activation of cytoplasmic caspase 1, which promotes maturation and the release of pro-inflammatory cytokine IL-1β [44, 59]. In 1970, Small and colleagues investigated the properties of cholesterol and identified monohydrate cholesterol crystals at 37°C and emphasized that extracellular cholesterol crystals are present in advanced atherosclerotic plaques. In addition, *in vitro* studies using lipid-filled macrophages that were transformed into foamy cells showed that following the accumulation of free cholesterol inside the cell, cholesterol monohydrate crystals are also formed. These findings showed that the accumulation of free and unesterified cholesterol can lead to the deposition of cholesterol crystals in the vessel wall [60]. In one study, cholesterol crystals were shown to be the main driver of intrinsic inflammation in spontaneously ruptured human aortic plaques, leading to the production of the pro-inflammatory interleukin-6 [61]. Studies have reported that cholesterol crystals can stimulate the NLRP3 inflammasome and release bioactive IL-1β and IL-18 in macrophages [62] .

Two types of inflammation mediated by cholesterol crystals have been proposed for cardiovascular disease:

1. Cholesterol crystals phagocytized by macrophages cause the release of pro-inflammatory cytokines in atherosclerotic lesions. Macrophages that have recognized ox-LDL through TRLs and scavenger receptors (SR) express pro-IL-1β. As a result, phagocytosis of cholesterol crystals induces the NLRP3 inflammasome complex and activates caspase 1, resulting in mature IL-1β [63, 64].
2. In advanced plaques, the formation of cholesterol crystals leads to the rupture of the fibrous cap of the plaque and thus initiates a systemic inflammatory response in the vessel wall [60].

## CONCLUSION

Considering that red blood cells (RBCs) can contribute to the excretion of cholesterol through the hepatobiliary system alongside HDL, and given that elevated cholesterol levels are a risk factor for the development and progression of atherosclerotic plaques, RBCs may play a protective role in cardiovascular health. However, when bleeding occurs within a plaque, RBCs that are trapped in the plaque environment, an environment rich in oxidant compounds, can rupture. The cholesterol released from these ruptured RBCs can significantly promote inflammatory reactions. The interaction between cholesterol and inflammatory processes plays a crucial role in the development and progression of atherosclerotic plaques. Cholesterol crystals act as Damage-Associated Molecular Patterns (DAMPs) and can trigger the activation of the NLRP3 inflammasome in macrophages. This activation of NLRP3 by cholesterol crystals leads to the activation of cytoplasmic caspase-1, which in turn promotes the maturation and release of the pro-inflammatory cytokine IL-1β. Ultimately, reducing cholesterol levels and controlling inflammatory processes can effectively prevent plaque instability and enhance the efficacy of drug treatments such as statins.

## LIST OF ABBREVIATIONS

Hb: Hemoglobin
RBCs: Red Blood Cells
CVD: Cardiovascular Diseases
ECM: Extracellular Matrix
SMC: Smooth Muscle Cell
LCAT: Lecithin Cholesterol Acyltransferase
ACS: Acute Coronary Syndrome
TLRs: Toll-like Receptors
